# Suicidality Associated With Deep Brain Stimulation in Extrapyramidal Diseases: A Critical Review and Hypotheses on Neuroanatomical and Neuroimmune Mechanisms

**DOI:** 10.3389/fnint.2021.632249

**Published:** 2021-04-08

**Authors:** Alessandra Costanza, Michalina Radomska, Guido Bondolfi, Francesco Zenga, Andrea Amerio, Andrea Aguglia, Gianluca Serafini, Mario Amore, Isabella Berardelli, Maurizio Pompili, Khoa D. Nguyen

**Affiliations:** ^1^Department of Psychiatry, Faculty of Medicine, University of Geneva (UNIGE), Geneva, Switzerland; ^2^Department of Psychiatry, ASO Santi Antonio e Biagio e Cesare Arrigo Hospital, Alessandria, Italy; ^3^Faculty of Psychology, University of Geneva (UNIGE), Geneva, Switzerland; ^4^Department of Psychiatry, Service of Liaison Psychiatry and Crisis Intervention (SPLIC), Geneva University Hospitals (HUG), Geneva, Switzerland; ^5^Department of Neurosurgery, University and City of Health and Science Hospital, Turin, Italy; ^6^Section of Psychiatry, Department of Neuroscience, Rehabilitation, Ophthalmology, Genetics, Maternal and Child Health (DINOGMI), University of Genova, Genova, Italy; ^7^Department of Psychiatry, IRCCS Ospedale Policlinico San Martino, Genoa, Italy; ^8^Mood Disorders Program, Tufts Medical Center, Boston, MA, United States; ^9^Department of Neurosciences, Mental Health and Sensory Organs, Suicide Prevention Center, Sant’Andrea Hospital, Sapienza University of Rome, Rome, Italy; ^10^Department of Microbiology and Immunology, Stanford University, Palo Alto, CA, United States; ^11^Tranquis Therapeutics, Palo Alto, CA, United States; ^12^Hong Kong University of Science and Technology, Hong Kong, China

**Keywords:** deep brain stimulation, suicide, neuroinflammation, extrapyramidal diseases, suicidal ideation (SI), suicidal behavior (SB), suicide attempt (SA), Parkinson’s disease

## Abstract

Deep brain stimulation (DBS) is a very well-established and effective treatment for patients with extrapyramidal diseases. Despite its generally favorable clinical efficacy, some undesirable outcomes associated with DBS have been reported. Among such complications are incidences of suicidal ideation (SI) and behavior (SB) in patients undergoing this neurosurgical procedure. However, causal associations between DBS and increased suicide risk are not demonstrated and they constitute a debated issue. In light of these observations, the main objective of this work is to provide a comprehensive and unbiased overview of the literature on suicide risk in patients who received subthalamic nucleus (STN) and internal part of globus pallidum (GPi) DBS treatment. Additionally, putative mechanisms that might be involved in the development of SI and SB in these patients as well as caveats associated with these hypotheses are introduced. Finally, we briefly propose some clinical implications, including therapeutic strategies addressing these potential disease mechanisms. While a mechanistic connection between DBS and suicidality remains a controversial topic that requires further investigation, it is of critical importance to consider suicide risk as an integral component of candidate selection and post-operative care in DBS.

## Introduction

Deep brain stimulation (DBS) has emerged as an effective therapy for patients with extrapyramidal disorders, particularly those with drug-refractory advanced Parkinson’s disease (PD). This neurosurgical procedure requires the implantation of electrodes to deliver electrical pulses from a neurostimulator into specific brain regions. Depending on various stimulatory parameters such as intensity (mA), desired frequency (Hz), and pulse duration (μs), DBS can differentially modulate neuronal activities to achieve desired therapeutic outcomes. Introduced more than two decades ago, this approach has been frequently employed to target the subthalamic nucleus (STN) and the internal globus pallidum (GPi) in PD (Pollak et al., 1993; Limousin et al., 1998). While STN has been the preferred DBS target with a greater impact on post-operative medication withdrawal (Moro et al., 2010; St George et al., 2015), GPi appears to be more programmable (easier to precisely target specific areas while avoiding off-targeting effects) due to its larger volume (Au et al., 2020). GPi DBS seems to have a more powerful anti-dyskinesia effect in comparison to STN DBS (Munhoz et al., 2014). Differences in efficacy between STN and GPi DBS have also been observed in studies of advanced PD with a particular emphasis on tremor, where the former provided enhanced motor improvement during the off-drug phase (Odekerken et al., 2016) and the latter resulted in less dyskinesia during the post-operative on-medication period (Tsuboi et al., 2020; Zhang et al., 2020). To date, DBS is the neurosurgical treatment of choice for drug-refractory PD with proven efficacy (Thobois et al., 2002; Herzog et al., 2003; Benabid et al., 2009; Schuepbach et al., 2013; Lezcano et al., 2016). The most profound effects of DBS are in the areas of rigidity (75% improvement) and akinesia (50% improvement), allowing a marked reduction in dopaminergic therapies, and consequentially, avoiding motor and non-motor complications (Benabid et al., 2009). While the generally high precision and efficacy of DBS represents a clinical success, its mechanism of action remains elusive (Denys et al., 2012; Chiken and Nambu, 2016; Herrington et al., 2016) with possible involvement in the modulation of primarily glutamatergic (STN-DBS) and GABAergic (GPi-DBS) neuronal circuits (Dostrovsky et al., 2000; Jakobs et al., 2019).

Some adverse outcomes in patients receiving this therapy have been reported. Notably, suicidal ideation (SI) and behavior (SB), including suicide attempts (SA) and completed suicides, have been observed but causal associations between DBS and increased suicide risk have not been established. Therefore, the primary goal of this work is to provide a concise and unbiased overview of studies on suicidality in patients receiving STN and GPi DBS. Additionally, putative mechanisms that might be involved in the development of suicidality in these patients as well as caveats associated with these hypotheses are introduced. Finally, we propose some clinical implications, including therapeutic strategies addressing these potential disease mechanisms to avoid the risk of suicidality in patients receiving DBS, with a particular emphasis on patient selection and neuropsychiatric post-operative care.

## Literature Overview

While comprehensive data on SI and SB rates of subjects with a co-morbid neurological condition or physical illness are often limited (Ostertag et al., 2019; Costanza et al., 2020d, i), clinical evidence of suicidality possibly associated with DBS treatment for PD subjects was first documented in a series of case reports (Doshi et al., 2002; Balash et al., 2007; Rodrigues et al., 2010). Additionally, analyses of initial cohorts of PD patients undergoing STN and GPi DBS in the early 2000s observed a notable rate of suicidality (1.5–4.6% for complete suicides, 0–6.1% for SA, and 12.5–16.6% for SI) despite the interventional success concerning motor symptom improvement (Berney et al., 2002; Houeto et al., 2002; Krack et al., 2003; Burkhard et al., 2004; Funkiewiez et al., 2004; Table 1). This phenomenon was particularly interesting because unlike other neurological conditions with well-established increases in suicide risk, such as multiple sclerosis and epilepsy (Costanza et al., 2015, 2020a), subjects with PD have a markedly lower risk in comparison to that of the general population (Li et al., 2018). Suicidality in patients with STN and GPi DBS was also reported in larger studies from 2006 to 2019 (Table 1), but with more varying frequencies of SI and SB compared to previous cohorts and it remained unclear whether this phenomenon was mechanistically related to DBS. This assertion was in particular challenged by two meta-analyses in a combined total of more than 10,000 subjects with extrapyramidal diseases, which yielded a substantially lower rate of suicide associated with DBS (~0.1–0.32%; Kleiner-Fisman et al., 2006; Appleby et al., 2007; Table 2). Conversely, a vast international multicenter study reported a 0.45% rate of suicide in more than 5,000 PD patients receiving DBS; interestingly, it was demonstrated that after adjustment for other demographic factors, the standardized mortality rate of DBS subjects appeared to be higher than that of the general population, especially in the first year after a DBS surgical procedure (Voon et al., 2008). While the incidences of SI and SB in patients who underwent DBS seemed to be significantly decreasing, probably due to improvement in candidate selection and postoperative follow-up (see paragraph 4), the debate was revived in 2019, when in a large retrospective case-controlled study by Giannini et al. (2019) in 534 PD patients who underwent bilateral STN DBS between 1993 and 2016, completed suicide and SA percentages were 0.75% and 4.11%, respectively. The observed suicide rate in the first postoperative year was higher than the expected National Observatory on Suicide Risks rate adjusted for age and sex and this rate remained similar over the second and third postoperative years (Giannini et al., 2019; Table 1). Two recent meta-analyses revealed increased rates of SI and SB (Xu et al., 2021), particularly in patients who underwent DBS compared to the general population (Du et al., 2020; Table 2). Two recent systematic reviews reaffirm the relevance of the problem of suicidality in DBS while insisting on the heterogeneity of the results and the extreme complexity in interpreting them (Berardelli et al., 2019; Zarzycki and Domitrz, 2020), while a third is more incisive in excluding any causal associations between DBS and suicidality (Shepard et al., 2019).

**Table 1:** Original studies on suicidality in patients who underwent deep brain stimulation (DBS).

References	Disease and sample size, *N*	Study design	Completed suicide, *N* (%)	SA *N* (%)	SI *N* (%)	Assessments methods
**STN DBS**						
Doshi et al. (2002)	PD 3	Case series	0	1 (33.3%)	N/A	UPDRS, clinical evaluation
Berney et al. (2002)	PD 24	Prospective (6-months follow-up)	0	0	3 (12.5%)	CAPSIT-PD, UPDRS HDRS, MADRS
Houeto et al. (2002)	PD 24	Retrospective	1 (4.6%)	0	4 (16.6%)	UPDRS, M.I.N.I., IOWA, SAS
Krack et al. (2003)	PD 49	Prospective (5-year follow-up)	1 (2%)	3 (6.1%)	N/A	UPDRS, Mattis DRS, Frontal-lobe function
Funkiewiez et al. (2004)	PD 77	Prospective (3-year follow-up)	1 (1.5%)	4 (5.7%)	N/A	Clinical records review, UPDRS, BDI, Mattis DRS, other neuropsychological scales
Smeding et al. (2006)	PD 99 cases, 39 controls	Case-control	0 cases 0 controls	1 (1%) cases 0 controls	N/A	Neurological, psychiatric, and neuropsychological battery
Balash et al. (2007)	PD 2	Case series	0	0	2 (100%)	Clinical evaluation
Soulas et al. (2008)	PD 200	Retrospective (9-year follow-up)	2 (1%)	4 (2%)	N/A	Clinical records review
Voon et al. (2008)	PD 5311	Retrospective 75 Worldwide centers survey	24 (0.45%)	48 (0.9%)	N/A	Neurological, psychiatric, and neuropsychological batteries
Porat et al. (2009)	PD 22	Prospective (1-year follow-up)	1 (4.5%)	1 (4.5%)	7 (31.8%)	Neurobehavioral Rating Scale, BPRS, BDI, Neuropsychiatric Inventory, Dopamine Deregulation, Work/Social Adjustment Scale
Rodrigues et al. (2010)	PD 3	Cases series	0	3 (100%)	N/A	UPDRS, LEDD, BDI
Soulas et al. (2011)	PD 41	Prospective (1-year follow-up)	1 (2.4%)	0	N/A	UPDRS, BDI, STAI, QoL
Umemura et al. (2011)	PD 180	Retrospective (7-year follow-up)	0	2 (1.1%)	N/A	UPDRS, clinical files review
Lhommée et al. (2012)	PD 63	Prospective	N/A	2 (3%)	N/A	M.I.N.I., Ardouin Scale
Børretzen et al. (2014)	ET 46	Prospective	1 (2%)	0	N/A	*Ad hoc* questionnaires
Giannini et al. (2019)	PD 534	Retrospective Case-control (9-year follow-up)	4 (0.75%)	22 (4.11%)	N/A	Clinical files review UPDRS part III, LEDD, Mattis DRS, Frontal Score, BDI
**STN and GPi DBS**						
Burkhard et al. (2004)	EMD 140	Retrospective (9-year follow-up)	6 (4.3%)	0	N/A	Clinical records review
Bernal-Pacheco et al. (2013)	PD 113	Retrospective (8-year follow-up)	0	0%	10 (11.5%)	BDI, behavioral features
Rocha et al. (2014)	PD 184	Prospective (50-month mean follow-up)	1 (0.5%)	0	0	UPDRS parts I-II, BDI, GDI
Boel et al. (2016)	PD 128	Prospective multicenter (3-year follow-up)	0	0	Low	M.I.N.I., MDRS, YMRS, HADS, NESDA
Buhmann et al. (2017)	EMD 123*	Retrospective (3.5-year follow-up)	1 (0.8%)	N/A	N/A	UPDRS, review of clinical files
**GPi DBS**						
Foncke et al. (2006)	DT 16	Retrospective (5-year follow-up)	2 (12.5%)	0	N/A	Dystonia scales, clinical files review
**STN-GPi DBS vs. BMT and STN vs. GPi DBS**						
Weintraub et al. (2013)	PD Phase I: 121 STN-GPi DBS, 134 BMT; Phase II: 147 STN DBS 152 GPi DBS	Randomized trial (6-month follow-up)	0%	0%	Phase I: 1.9 STN-GPi DBS, 0, 9% BMT; Phase II: 1.5% in STN DBS vs. 0.7% in GPi DBS	UPDRS part I
**STN DBS vs. BMT**						
Witt et al. (2008)	PD 78 DBS 78 BMT	Randomized multicenter	1 (1.3%) DBS 0 BMT	N/A	N/A	BDI, MADRS
Strutt et al. (2012)	PD 17 DBS, 22 BMT	Prospective	1 (5%) DBS 0 (BMT)	0 DBS 0 (BMT)	N/A	BDI, STAI
Deuschl et al. (2013)	PD 124 DBS, 127 BMT	Randomized trial (2 -year follow-up)	2 (1.6%) DBS 0 BMT	2 (1.6%) DBS 0 BMT	1 (0.8%) DBS 0 BMT	Neurological, psychiatric, and neuropsychological battery
Lhommée et al. (2018)	PD 124 DBS, 127 BMT	Case-control (2-year follow-up)	2 (1.6%) DBS 1 (0.8%; BMT)	4 (3%) DBS 5 (4%) BMT	4 (3%) DBS 5 (4%) BMT	Ardouin Scale, BDI, Starkstein Apathy Scale

*BDI, beck depression inventory; BMT, best medical treatment; BPRS, brief psychiatric rating scale; CAPSIT-PD, core assessment program for surgical interventional therapy for PD; DBS, deep brain stimulation; DT, dystonia; EMD, extrapyramidal movement disorder; ET, essential tremors; GPi, globus pallidus internus; HADS, hospital anxiety and depression scale; HDRS, hamilton depression rating scale; IOWA, iowa rating scale of personality changes; LEDD, L-dopa equivalent dose; MADRS, montgomery asberg depression rating scale; Mattis DRS, mattis dementia rating scale; M.I.N.I., mini international neuropsychiatric inventory; N/A, unavailable; NESDA, Netherlands study on depression and anxiety interview; PD, Parkinson’s disease; SA, suicidal attempt, SAS, social adjustment scale; SI, suicidal ideation; STN, subthalamic nucleus; UPDRS, unified pd rating scale; YMRS, young mania rating scale*.

**Table 2:** Meta-analysis on suicidality in patients who underwent DBS.

References	Disease and sample size	Study design	Completed suicides	SA	SI
Kleiner-Fisman et al. (2006)	PD 971	Meta-analysis (1993–2004)	0.1%	0.7%	N/A
Appleby et al. (2007)	EMD 10399	Meta-analysis (1996–2005)	0.16–0.32%	0.3–0.7%*	0.3–0.7%*
Du et al. (2020)	PD	Meta-analysis (1990–2019)	(a) DBS vs. drug therapy: No significant differences; (b) DBS vs. general population: Significant increased risk of suicide and/or SI (OR = 2.839–3.927, *p*< 0.0001).
Xu et al. (2021)	PD	Meta-analysis (all eligible studies till 2019)	1%	1%	4%

DBS, Deep Brain Stimulation; EMD, extrapyramidal movement disorder; N/A, unavailable; PD, Parkinson’s Disease; SA, suicidal attempt, SI, suicidal ideation. *SA and SI considered together as “suicidality”.

Besides studies that focus on DBS at the STN or the STN and GPi, an examination of suicidality in patients receiving only GPi DBS treatment has been scarcely reported. In this regard, complete suicides (12.5%) were documented in a small cohort of patients with dystonia (Foncke et al., 2006). A comparative analysis of SI incidence in PD patients receiving DBS at two different neurological targets (GPi vs. STN) showed a markedly lower rate in the GPi group (Weintraub et al., 2013; Table 1). It was postulated that GPi DBS might be protective against the development of depression or SI and SB because it is comparable to STN DBS concerning motor outcomes but does not require postoperative reduction of dopaminergic medications as is usually the case for STN DBS (Weintraub et al., 2013). Moreover, GPi DBS might provide better protection against the development of depression or SI and SB as this treatment seemed to result in greater relief from psychiatric symptoms (Liu et al., 2014; Negida et al., 2018). A meta-analysis found that stimulating STN and GPi were equally effective at improving motor symptoms and dyskinesias; however, there would be has been discrepancy as to whether the cognitive, behavioral, and mood symptoms were affected differently between the two targets (Combs et al., 2015). This issue needs further research (Combs et al., 2015).

Greater SI and SB incidences in case-control studies among PD patients treated with DBS compared to PD patients treated with best medical treatment (BMT) were reported (0–5% for complete suicides, 0–3% for SA, and 0–3% for SI; Witt et al., 2008; Strutt et al., 2012; Deuschl et al., 2013; Weintraub et al., 2013; Lhommée et al., 2018). However, these studies do not yield uniform results. In the cohort of Lhommée et al. (2018), it was shown that there was a two-fold increase in complete suicides (1.6% vs. 0.8%) in patients with DBS compared to patients who received BMT, but SA and SI were less frequent in former patients than in latter (3% vs. 4%; Table 1).

To date, the topic of suicidality and DBS remains controversial due to differences in clinical assessment methods, study types, sample sizes, and patient characteristics. Nevertheless, these reports highlight possibly DBS-associated suicidality as an important phenomenon that deserves further examination and clinical attention.

## Hypotheses on Mechanisms for Suicidality Associated with DBS

### Neuroanatomical Circuit Dysfunctions

**Figure 1:**
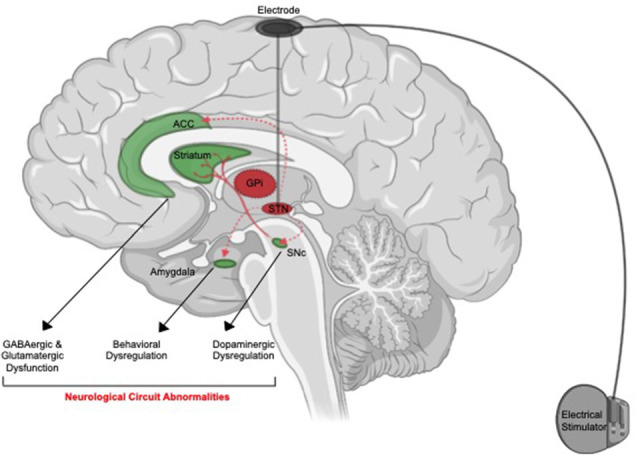
Putative neuroanatomical mechanisms of suicide associated with deep brain stimulation (DBS). Off-target stimulation of brain regions connected with anterior cingulate cortex (ACC, amygdala) or adjacent to (SNc) the suicide risk in patients who received subthalamic nucleus (STN) might cause induction of neurological pathways associated with suicide. Activation of the ACC might cause a disturbance in GABAergic and glutamatergic signaling. Activation of the amygdala might result in behavioral abnormalities. Activation of SNc and dorsal striatum circuit can result in dopaminergic dysfunction. These neurobiological changes might translate into elevated psychosocial distress and potentiate suicidal ideation (SI) and suicidal behavior (SB).

While many neurobiological factors have been associated with suicide (Hawton and van Heeringen, 2009; Turecki et al., 2012; Costanza et al., 2014; van Heeringen and Mann, 2014; Costanza et al., 2020j), little is known about the neuro-etiology of this phenomenon in the context of DBS. The primary hypothesis on putative neurobiological mechanisms of suicide after DBS revolves around the anatomical regions that might be undesirably targeted by this treatment, notably those structurally adjacent or functionally related to STN or GPi (14; Figure 1).

Specifically, STN stimulation can modulate dopamine release from the substantia nigra pars compacta (SNc) in animal studies (Shimo and Wichmann, 2009). In light of the critical involvement of dysfunctional dopaminergic signaling in PD patients, DBS might further disrupt the dopaminergic system, potentiating SI or SB. Mechanistically, since dopamine has been shown to drive impulse control disorders (Ahlskog, 2011), the dysregulated release of this neurotransmitter might consequently increase the risk of SI (Costanza et al., 2014). In fact, dysregulated activation of dopamine signaling, particularly in the dorsal striatum (the main target of SNc dopaminergic projections), has been linked to suicide (Fitzgerald et al., 2017). Consistent with this report, a recent study has demonstrated a correlation between reduced dopamine transporter availability and increased hopelessness scores in all bilateral striatal areas of subjects with major depressive disorders (MDD; Pettorruso et al., 2020). Last but not least, it is also worth noting that a rapid post-DBS reduction in dopamine therapy might cause a general hypodopaminergic state in the brain and the unmasking of depression and SI in PD patients (Berardelli et al., 2019).

Another important brain region that is affected by DBS at the STN is the amygdala, which regulates emotions and aggressive behaviors (Accolla et al., 2016). Since abnormalities in functions and structure of this brain region have been associated with SA in subjects with neuropsychiatric diseases (Spoletini et al., 2011; Wang et al., 2020), possibly off-target stimulation of the amygdala might drive suicide risk in DBS subjects. Increased functional connectivity at this neuroanatomical region has been linked to increased SI and SA in patients with MDD (Kang et al., 2017; Alarcón et al., 2019). Association between the self and death in an exploratory study of the neurobiological origins of SI has also been attributed to amygdala activation (Wei et al., 2018; Ballard et al., 2020). Mechanistically, activation of the amygdala can exacerbate dopaminergic dysfunction (Lai and Chang, 2019) and consequently potentiating suicide risk as described above. Enhanced amygdala connectivity has also been linked to excessive glutamatergic-induced neuro-excitotoxicity and increased suicide risk (Sequeira et al., 2009; Cabrera et al., 2019; Ousdal et al., 2019).

It was also hypothesized that DBS-induced modulation of connections between the STN and the prefrontal cortex areas and mid-brain serotonin neurons may be implicated in mood-related changes and subsequent increased suicide risk (Temel et al., 2005).

Similarly, STN connectivity with frontal cortical regions such as the anterior cingulate cortex (ACC) is highly relevant in the context of suicide (Brunenberg et al., 2012). The ACC is a complex cortical region that modulates emotional behaviors and abnormalities in its function are the basis of the development of several mood disorders (Drevets et al., 2008). In suicidal subjects, ACC functional activity was enhanced (Minzenberg et al., 2016). ACC activation also results in increases in glutamate/GABA expression (Zhao et al., 2018; Lewis et al., 2020). As a result of this, bystander-stimulation of the ACC during DBS therapy might drive these neurotransmitter disturbances, which have been linked to the increased risk for suicide (Sequeira et al., 2009; Cabrera et al., 2019).

Despite these speculative mechanisms, it is worth noting that contradictory findings have indicated a reduction in functional connectivity at the amygdala (Johnston et al., 2017; Wang et al., 2020) and ACC (Huber et al., 2020) in patients with SI/SA. Therefore, definitive proofs for the involvement of the amygdala and/or ACC off-targeting in the development of DBS-associated suicidality have yet to be formally demonstrated.

### Aberrant Immunological Activation

**Figure 2:**
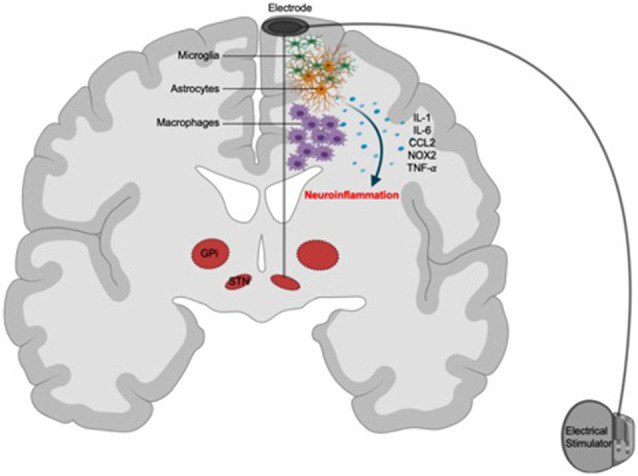
Putative immunological mechanisms of suicide associated with DBS. Surgical implantation of neuroelectrodes might cause immunoreactivity against these devices. Focal activation of recruited peripheral macrophages and brain resident microglia and astrocytes (glial scarring) in response to the electrodes triggers the release of inflammatory mediators, such as IL-1, IL-6, CCL2, NOX2, and TNF-α, resulting in neuroinflammation and subsequent aggravation of psychosocial risk factors for suicide.

The speculative involvement of the immune system in suicide associated with DBS stems from emerging evidence that links neuroinflammatory responses, orchestrated by microglia and macrophages, to both of these phenomena (Figure 2).

Microglia mature from a yolk sac progenitor and take residence in the nervous tissues while macrophages originate from the bone marrow and could be recruited to other tissues in various pathological contexts (Thion and Garel, 2020). Despite their ontological differences, the primary function of these innate immune cells is to patrol the body during homeostatic development to support tissue debris clearance (Butovsky and Weiner, 2018; Kierdorf et al., 2019; Tay et al., 2019). However, these cells are also able to respond to pathogens, noxious environmental insults, and surgical implants. Upon recognition of these foreign stimuli, microglia and macrophages elicit a robust inflammatory reaction, characterized by the release of cytokines and chemokines to amplify and sustain this response. While the intended function of this reactive inflammation is to protect the body from invading micro-organisms, its maladaptation could result in unresolved tissue damage and ensuing pathology.

Microglial reactivity in DBS has been observed in animal studies. For instance, STN implantation of stainless-steel electrodes triggered a localized microglial activation and sustained memory impairment (Hirshler et al., 2010). Similarly, cortical implants also resulted in chronic gliosis of microglial and astrocytic origins (Griffith and Humphrey, 2006; Rosskothen-Kuhl et al., 2018). Consistent with these observations, another study revealed a more elaborated spatial and temporal neuroinflammatory cascade, characterized by neuronal and progenitor cell loss, axonal and myelin reassembly, microglia and astrocyte reactivity, and pericyte deficiency-driven blood-brain barrier disruption, both acutely and chronically around implanted devices (Wellman et al., 2019). Interestingly, the density of neural tissues and the implanted probes as well as the size and fixation method of the implants play an important role in determining the extent of microglia and astrocyte reaction with regions with high-density probes exhibiting more pronounced neuroinflammation (Thelin et al., 2011; Lind et al., 2012) while the numbers of implanted devices did not appear to affect glial cell scarring (Lind et al., 2013). Furthermore, infiltrating macrophages have recently been shown to be the primary orchestrators of neuroinflammatory reaction to cortical implanted electrodes (Ravikumar et al., 2014). In this study, the authors highlight the hallmark accumulation of infiltrating macrophages at the implant sites and the positive correlation between their density and neuronal damage. Therefore, macrophages and microglia might cooperate to induce a biphasic neuroinflammatory and degenerative response to a chronically implanted device (Potter et al., 2012; Giordano et al., 2020). Corroborating evidence from studies on post-mortem brain tissues in humans also confirmed these preclinical findings. In a pediatric status dystonicus case study, evidence of gliosis and multinucleated giant cells (macrophage aggregates) was observed along the trajectories of the implanted electrodes (Kronenbuerger et al., 2015). Larger case series in PD patients also revealed extensive gliosis and macrophage accumulation in the majority of subjects undergoing DBS (Vedam-Mai et al., 2018). Notably, gliosis is associated with septicemia in deceased subjects (Kronenbuerger et al., 2015) but does not correlate with the duration of the DBS regimen (Vedam-Mai et al., 2018). Along with studies in animals, these findings suggest that neuroinflammation occurs during DBS and might represent a reaction against implanted neuroelectrodes, rather than against the DBS treatment itself (Amorim et al., 2015; Hadar et al., 2017; Lopez-Cuina et al., 2018).

Similar to DBS, microglia and macrophages have been implicated in a stress-diathesis paradigm of SI and SB (Baharikhoob and Kolla, 2020). It is postulated that suicide results from a complex interplay between psychosocial and biological stressors during which biological stimuli, i.e., macrophage and microglial mediated-neuroinflammation, act to aggravate the non-biological determinants of suicide. In support of this hypothesis, microgliosis was observed in post-mortem brain tissues of suicide subjects (Steiner et al., 2008; Schnieder et al., 2014; Torres-Platas et al., 2014; Cabrera et al., 2019). Markers of macrophage-mediated systemic inflammation, such as IL-1, IL-6, and TNF-α (Steiner et al., 2013), as well as those of localized microglia-derived neuroinflammatory responses, such as IL-6 and NOX2, are elevated in suicide subjects (Ganança et al., 2016; Schiavone et al., 2016). Furthermore, suicide is associated with neuroinflammatory signatures in selective brain regions, suggesting widespread inflammation is not necessary for the precipitation of SB (Serafini et al., 2020). Interestingly, in depressed suicides, microglia expression of quinolinic acid was elevated (Steiner et al., 2011). Macrophage infiltration was also documented in the brain tissues of these subjects, evident by increased expression of the surface protein CD45 and the soluble chemokine CCL2 (Brisch et al., 2017). Last but not the least, nonsteroidal anti-inflammatory drugs that could effectively curb neuroinflammation can reduce SI in humans (Lehrer and Rheinstein, 2019), further supporting the role of glial-cell-mediated neuroinflammation in the potentiation of suicide risk.

Mechanistically, these microglia and macrophage-derived neuroinflammatory mediators are thought to interfere with homeostatic serotonergic and glutamatergic transmission by the metabolic pathway of indoleamine 2,3-dioxygenase (IDO; Suzuki et al., 2019; Baharikhoob and Kolla, 2020; Serafini et al., 2020). In this prominent hypothesis on the inflammatory origin of suicide, augmented inflammatory milieu in the specific brain regions that are produced by microglia can activate IDO-dependent catabolism of tryptophan to generate kynurenine. This metabolite in turn promotes the development of neurotoxic microglia, which can produce quinolinic acid and consequentially trigger glutamatergic/NMDA-receptor-dependent neurotoxicity. Alternatively, tryptophan degradation could also result in reduced serotonin production, which has been implicated in mood disorders and suicide (Pompili et al., 2017).

Given the collective implications of neuroinflammation elicited by macrophages and microglia in both DBS and suicide, it is plausible to envision that suicide associated with DBS might result from unregulated regional reactivity of these cells to the implanted device. Notably, innate immune cells in these subjects might also be more susceptible to eliciting dysregulated inflammation due to their underlying neurological conditions, potentiating the risk for psychosocial stress-driven suicide.

Nevertheless, both experimental and clinical data to formally support this hypothesis are currently lacking. Due to the lack of validated animal models of suicide and mood disorder, future studies that utilize microglial PET tracers (Cavaliere et al., 2020) in patients with DBS would be required to provide a definitive answer to this topic of immense clinical and scientific interest.

## Clinical Implications

The selection of the candidate patient to undergo DBS is the most effective means of avoiding psychiatric complications, including suicidality development, following operation (Lang and Widner, 2002; Rodriguez et al., 2007; Pollak, 2013; Boel et al., 2016). In this regard, the presence of a multidisciplinary team consisting of a neurologist, a psychiatrist, and a psychologist is of critical importance for a comprehensive evaluation of the patient’s suitability for DBS treatment. Eligibility guidelines for DBS include patients diagnosed with extrapyramidal diseases that are either refractory to conventional medications (i.e., inconsistent or marginal responses to medical treatments that result in fluctuating motor symptoms and/or dyskinesia) or chronically/severely suffering from medication-related side effects. Besides these inclusion criteria, disabling and active psychiatric disturbances, history of SI/SB, past diagnoses of unipolar/bipolar affective or psychotic disorders represent contraindications to DBS. Furthermore, even in the absence of major psychiatric disturbances, attention must be paid to symptoms of emotional lability, apathy, impulsivity, and irritability/anger. In these situations, the patients must be fully treated for such minor psychiatric issues to proceed with DBS operation, if DBS treatment is considered necessary. It’s also worth noting that the physicians should inform the patients about the potential development of psychiatric disturbance after DBS surgery even if they had no previous history of these symptoms. Other exclusion criteria for DBS include the presence of moderate/severe cognitive impairment as well as other medical comorbidities. DBS therapy is also not recommended when insufficient motivation or unrealistic expectations are expressed by the patient or their family members. The patients should be fully aware that DBS treatment might require series of surgical and medication adjustments and thus, patience and significant time commitment are required. Lastly, the patients should be realistic about the efficacy of this treatment as it is not the ultimate cure for extrapyramidal diseases and only results in meaningful therapeutic outcomes (Lang and Widner, 2002; Rodriguez et al., 2007; Pollak, 2013; Boel et al., 2016).

After the DBS, a consistent post-operative follow-up with a multidisciplinary team of physicians, particularly in the first few years after DBS surgery, is recommended (Lang and Widner, 2002; Rodriguez et al., 2007; Pollak, 2013; Boel et al., 2016). During such routines, detailed clinical interviews with the patients and their caregivers should be conducted. To assess the progression of extrapyramidal diseases and various parameters the patients’ psychiatric/psychological conditions, standardized assessment scales, including the United Parkinson’s Disease Rating Scale (UPDRS; Poewe, 2009), the Montgomery–Åsberg Depression Rating Scale (MADRS; Williams and Kobak, 2008), the Columbia-Suicide Severity Rating Scale (C-SSRS; Posner et al., 2011), the Ardouin Scale of Behavioral in Parkinson’s Disease (ASBPD; Rieu et al., 2015) for psychiatric assessment related to hypodopaminergic (as apathy) and hyperdopaminergic (as dysregulated impulse control) and non-motor fluctuations, and the Austin CEP Interview for expectation assessment (adapted from the field of surgical epilepsy; Wilson et al., 1999), could be employed. Additionally, since increased or *de novo* cognitive impairment after DBS surgery could influence psychiatric complications, comprehensive neurocognitive assessment should also be included as an integral part of postoperative follow-up. Collectively, the findings from these assessments provide a comprehensive snapshot of the post-operative state of the patients, which could be compared with their pre-operative status so that appropriate interventions could be devised.

Psychiatric post-operative care involves both psychopharmacological adjustments and psychotherapeutic interventions, such as the recommended cognitive-behavioral therapies (CBT). Psychotherapeutic interventions, which are particularly relevant in the context of suicide prevention (Costanza et al., 2020k, 2021), could also address other specific features of PD patients receiving DBS, such as frustration due to “disproportionate or unrealistic expectations” related to DBS (Wrench et al., 2004), and the occurrence of a scenario named “burden of normality” (often detected in patients whose chronic and disabling disease could be rapidly and drastically improved; thus paradoxically exposing them to difficulties in restoring normal life; Wilson et al., 2001, 2004; Gilbert, 2012). In this regard, the two constructs of demoralization and Meaning in Life (MiL) have recently received clinical attention as psychotherapeutic targets for these PD patients, similarly to those who experienced a severe somatic disease and underwent a disjointed “before” and an “after” existential experience (Costanza et al., 2019, 2020c,f). These two constructs are intimately associated with each other as loss of MiL is one of the constituting components of demoralization (Chytas et al., 2019; Costanza et al., 2020e). Furthermore, MiL and demoralization are two important resilience and risk factors, respectively, for the development of suicidality (Costanza et al., 2020b,g,h). Other promising psychotherapeutic targets include the two dimensions of Interpersonal Theory of Suicide (IPTS), such as the feeling of “perceived burdensomeness” and “thwarted belongingness” (Baertschi et al., 2017, 2018a,b). The interpersonal nature of IPTS, in particular, can address psychoeducational needs for both patients and their family members/care-givers (Costanza et al., 2018).

Besides these interventions, strategies aiming at correcting neuroanatomical and neuroimmunological dysfunctions could be devised to address the possible involvement of these pathways in the development of suicidality in patients receiving DBS. For instance, given the acute reduction in dopamine therapy in DBS patients after undergoing this treatment, dosing could be adjusted accordingly to avoid undesirable effects of a DBS induced-hypodopaminergic state (Berardelli et al., 2019). Targeting ACC functional connectivity defects that are associated with suicide might also be specifically achieved with the emerging use of ketamine and esketamine (Chen et al., 2019a, b). Additionally, strategies to suppress neuroinflammation have also been attempted. Specifically, experimental studies have revolved around the development of surgical innovations to mitigate the response of the immune system to the DBS implants. As such, different materials, surface structure, shapes, size, density, and fixation methods of the implant devices might be critical to minimize microglial reactivity (Thelin et al., 2011; Lind et al., 2012, 2013; Eles et al., 2017; Golabchi et al., 2019). Furthermore, adjunct treatment with anti-inflammatory agents systemically (Yuan et al., 2019) or locally properties (Zhong and Bellamkonda, 2007; Gutowski et al., 2015; Liu et al., 2017) also presents an attractive therapeutic roadmap.

## Conclusion

In summary, while it remains elusive whether and to what extent neuroanatomical and/or immunological mechanisms contribute to suicide associated with DBS, this phenomenon represents an unmet medical need. Besides the novel therapeutic insights stemming from these mechanistic postulates and in line with the current shift in healthcare toward predictive/precision medicine rather than secondary preventative methods (Amerio et al., 2020), more consensual and multi-disciplinary guidelines (including neurosurgical, psychiatric, and psychological interventions) both for DBS candidate selection and for post-operative follow-up of patients undergoing DBS are of critical importance.

## Data Availability Statement

The original contributions presented in the study are included in the article, further inquiries can be directed to the corresponding author.

## Author Contributions

AC, MR, AAm, AAg, and FZ contributed to the conception of the work, researched the literature, and drafted the primary manuscript. GS, MA, GB, IB, and MP contributed to the conception of the work, carefully revised the manuscript, and provided the intellectual impetus. KDN supervised all steps of the work, revised, finalized, and edited the final version of the manuscript. All authors contributed to the article and approved the submitted version.

## Conflict of Interest

KN is the scientific founder of Tranquis Therapeutics, a neuroimmunology company that develops immunotherapies for neurological diseases. The remaining authors declare that the research was conducted in the absence of any commercial or financial relationships that could be construed as a potential conflict of interest.
